# The Steady State Characteristics of Multicomponent Diffusion in Micro- and Mesoporous Media for Adsorbable and Nonadsorbable Species

**DOI:** 10.3390/membranes12100921

**Published:** 2022-09-23

**Authors:** Katarzyna Bizon, Dominika Boroń, Bolesław Tabiś

**Affiliations:** Faculty of Chemical Engineering and Technology, Cracow University of Technology, 31-155 Kraków, Poland

**Keywords:** meso- and microporous solids, multicomponent complex diffusion, steady states, modeling, algorithm

## Abstract

The study addresses one of the fundamental issues in the mathematical modeling and quantitative process analysis of complex multicomponent diffusion in meso- and microporous materials. The model presented here incorporates combined molecular diffusion, Knudsen diffusion, viscous flow, and surface diffusion. A methodology and algorithm for the determination of steady states of such complex diffusive processes are proposed. The adopted form of the surface diffusion model does not require the calculation of the thermodynamic factor matrix. The method was verified by comparing the profiles of the state variables with those obtained from the dynamic model for sufficiently long diffusion times. The application of the method is illustrated for two diffusion processes involving three components. In the first one, all components are subject to adsorption. In the other, one gaseous component is an inert and is therefore not adsorbed and does not participate in surface diffusion. It is shown that the presence of inerts as well as their number does not impede the application of the proposed algorithm for the determination of steady states.

## 1. Introduction

The literature on diffusive mass transport in bulk phases and porous materials is extremely rich. A review of articles published up to nearly the end of the 20th century on the application of the Maxwell–Stefan formalism for the quantitative analysis of such processes can be found among others in the work of Krishna and Wesseling [1]. Processes involving porous solids play an unquestionable role in numerous operations encountered in chemical engineering and technology [2,3,4], in the fuel industry [5,6,7], in chemical analytics [8], and in the food industry [9]. These processes include chemical reactions in porous catalysts [2,3,4], adsorptive separation processes [10,11], and chromatographic analysis. An important application of porous materials are membrane multifunctional reactors [12,13,14].

**Figure 1:**
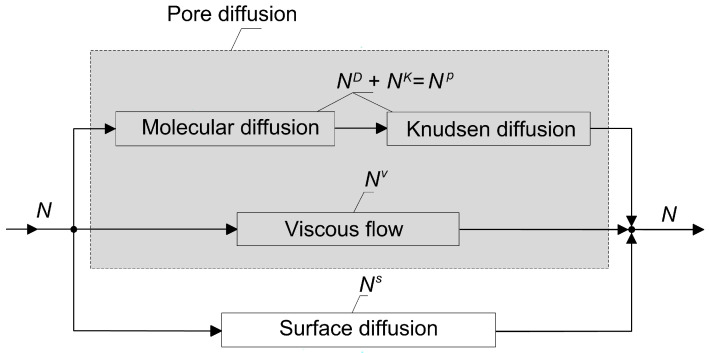
Constituent elements of resistance to mass transport in porous materials with indicated molar flux densities.

The quantitative description of diffusive mass transport in porous media is much more complex than the description of diffusion in fluids [15]. It involves the interaction of the solid with the molecules of the diffusing substance. This interaction, generally speaking, depends on the ratio of the mean free path of the molecules to the pore diameter, and on the relationship between the sizes of the molecules and the pores. These two dependences are superimposed on the thermodynamic features of the process, i.e., the interphase equilibrium and the condensation temperatures of the diffusing substances. This provides a great variety of mechanisms of mass transport in porous solids. A conceptual diagram illustrating different mechanisms of mass transport in porous materials and the relationship among them is shown in Figure 1.

The diffusive processes that occur in the particles of adsorbents involve transient mass transport. Membrane separation operations generally occur under steady-state conditions. A fundamental property of mass transport processes in porous materials is the diffusion characteristics; dynamic or steady-state, respectively. The determination of such characteristics requires knowledge of both the mathematical models and the methods of solving the relevant equations describing these processes.

Today, the dusty gas model [16,17,18] is usually used for quantitative description of diffusion in the pores of solids, while the generalized Maxwell–Stefan formalism [19,20,21,22,23] is most commonly used to model multicomponent mass transport by surface diffusion. It can be observed that both in the literature cited here and in other works where such complex diffusion is discussed, models describing simultaneous pore and surface diffusion are presented in such a way that only the total molar flux densities **N***_tot_* can be calculated [21,24,25,26]. In the following, it is shown how to calculate the molar flux densities under steady-state conditions separately for diffusion in pores **N***^p^* and via surface diffusion **N***^s^*. Obviously, this provides the possibility of calculating **N***_tot_* = **N***^p^* + **N***^s^*. Furthermore, it establishes a framework for theoretical analysis of the contribution of **N***^p^* and **N***^s^* fluxes depending on the conditions under which diffusion takes place.

The primary objective of this work consists in proposing a mathematical model describing steady-state multicomponent complex diffusion in porous media, providing a general method for the determination of steady states and then illustrating it using two ternary gas mixtures as examples. In the proposed diffusion model, the thermodynamic factors Γ*_ij_* do not occur, and therefore, there is no need to search for inverse functions of the sorption isotherms *q_i_* = *q_i_* (**p**). Matrix notations are used in the numerical algorithms, which makes them easily extendable to any number of gaseous components within the mixtures, both adsorbable and non-adsorbable.

## 2. Materials and Methods

### 2.1. Description of the Dynamics of Complex Multicomponent Diffusion

Let us assume that in a microporous medium with a geometry defined by the shape factor *m*, diffusion involving *K* components occurs under isothermal conditions. The mechanisms contributing to mass transport in a complex diffusion process are: molecular diffusion and Knudsen diffusion in pores, viscous flow, and surface diffusion. The mathematical model of such diffusion for unsteady conditions is given by the system of equations [24,25]:

(1) $$\frac{\varepsilon }{RT}\frac{\partial p}{\partial t}+(1-\varepsilon )\rho_{s}\frac{\partial q}{\partial t}=-\frac{1}{z^{m}}\frac{\partial }{\partial z}\left[z^{m}N^{p}+z^{m}(1-\varepsilon )N^{s}\right]$$

where

(2) $$p={\left[p_{1},p_{2},\ldots ,p_{K}\right]}^{T},\;q={\left[q_{1}(p),q_{2}(p),\ldots ,q_{K}(p)\right]}^{T}$$

(3) $$N^{p}={\left[N_{1}^{p},N_{2}^{p},\ldots ,N_{K}^{p}\right]}^{T},\;N^{s}={\left[N_{1}^{s},N_{2}^{s},\ldots ,N_{K}^{s}\right]}^{T}$$

The system of Equation (1) represents the relationship between the changes in the amount of mass in the gas phase and those in the solid, and the molar flux densities in the pores **N***^p^* and by surface diffusion **N***^s^*. In the first term of the right-hand side of Equation (1), the porosity of the solid ε does not appear, as this quantity is incorporated in the definitions of the effective diffusion coefficients. The mathematical model (1) was developed assuming local thermodynamic equilibrium between the adsorbed phase and the bulk phase in the pores at each value of the spatial coordinate *z*. If the fugacity coefficients of the gas phase components *f_i_* are close to unity, then the aforementioned equilibrium is given by the following relationships:

(4) $$\mu_{i}^{s}=\mu_{i}^{g}=\mu_{pi}^{\circ }+RT\ln\frac{p_{i}}{p^{\circ }},\;(i=1,\;2,\ldots ,K)$$

Surface diffusion occurs when the adsorption of components on the solid surface takes place. The sorption isotherms in the *K*-component system are generally defined as:

(5) $$q_{i}=f_{i}(p)\;\mathrm{or}\;\theta_{i}=g_{i}(p)\;,\;T=\mathrm{const},\;(i=1,\;2,\ldots ,K)$$

where a fractional surface occupancy of component *i* is defined as:

(6) $$\theta_{i}=\frac{q_{i}}{q_{i}^{*}},\;(i=1,\;2,\ldots ,K)$$

In light of the assumption of the thermodynamic equilibrium mentioned above, that is, when the concentrations in the gas phase and on the surface of the solid are unambiguously dependent on each other, the derivatives of surface concentrations with respect to time appearing in Equation (1) can be written as:

(7) $$\frac{\partial q}{\partial t}=\frac{dq}{dp}\frac{\partial p}{\partial t}=J(p)\frac{\partial p}{\partial t}$$

The term **J**(**p**) is the derivative of vector **q** with respect to vector **p**. It is therefore a Jacobi matrix, which is calculated using the information on sorption isotherms (5). Finally, the mathematical model of multicomponent complex diffusion can be expressed as follows:

(8) $$\left[\frac{\varepsilon }{RT}I+(1-\varepsilon )\rho_{s}J(p)\right]\frac{\partial p}{\partial t}=-\frac{1}{z^{m}}\frac{\partial }{\partial z}\left[z^{m}N^{p}+z^{m}(1-\varepsilon )N^{s}\right]$$

where Jacobi matrix **J** is defined as:

(9) $$J(p)=\left[\begin{array}{cccc} \frac{\partial q_{1}}{\partial p_{1}} & \frac{\partial q_{1}}{\partial p_{2}} & ..... & \frac{\partial q_{1}}{\partial p_{K}}\\ ..... & ..... & ..... & .....\\ \frac{\partial q_{K}}{\partial p_{1}} & \frac{\partial q_{K}}{\partial p_{2}} & ..... & \frac{\partial q_{K}}{\partial p_{K}} \end{array}\right]$$

According to the dusty gas model [16,27], the vector of molar flux densities in the pores **N***^p^*, appearing in Equation (1), is given by the equation:

(10) $$N^{p}=-\frac{1}{RT}{\left[B^{p}\right]}^{-1}\frac{dp}{dz}-\frac{B^{e}}{RT\eta }\frac{dp}{dz}\cdot {\left[B^{p}\right]}^{-1}\cdot E\cdot p$$

where the elements of matrices **B***^p^*, **E** and **p** are defined according to Equations (11) and (12):

(11) $$B^{p}={\left\{B_{ij}^{p}\right\}}_{K\times K}={\left[\begin{array}{cc} \sum_{\begin{array}{l} k=1\\ k\neq i \end{array}}^{K}\frac{y_{k}}{D_{ik}^{e}}+\frac{1}{D_{Kn,i}^{e}}; & \left(i=j\right)\\ -\frac{y_{i}}{D_{ij}^{e}}; & \left(i\neq j\right) \end{array}\right]}_{K\times K}$$

(12) $$E\cdot p=\left[\begin{array}{c} \frac{p_{1}}{D_{Kn,1}^{e}}\\ ....\\ \frac{p_{K}}{D_{Kn,K}^{e}} \end{array}\right]$$

and

(13) $$y={\left[y_{1},y_{2},....,y_{K}\right]}^{T},\;y_{i}=\frac{p_{i}}{p},\;p=\sum_{i}p_{i}$$

The effective molecular and Knudsen diffusion coefficients are calculated respectively as:

(14) $$D_{ij}^{e}=\frac{\varepsilon }{\tau }D_{ij}$$

(15) $$D_{Kn,i}^{e}=\frac{\varepsilon }{\tau }D_{Kn,i},\;D_{Kn,i}=\frac{d_{p}}{3}\sqrt{\frac{8RT}{\pi M_{i}}}$$

It is believed [28] that the coefficients of molecular diffusion in pores *D_ij_* may be calculated according to the correlation of Fuller and co-authors [29,30].

It is worth mentioning here that the method of estimating the values of Knudsen diffusion coefficients was the subject of detailed discussion in the literature [31,32,33,34,35,36,37,38,39]. Krishna pointed out that the Knudsen diffusion coefficients can be correlated with Henry constant He for adsorption [28]. They increase with increases in this constant. In the case of many industrial processes such as adsorption or chemical reactions in the porous catalysts, the dominant mechanism of mass transport in the pores may be Knudsen diffusion [2,3,4,5,27]. Therefore, the contribution of this transport mechanism should be present in the general model (8). Knudsen diffusion is said to occur when collisions of molecules with pore walls are more frequent than with each other. The occurrence of the Knudsen mechanism is favored by low pressures. This is because then the mean free path of molecules increases.

The parameter B*^e^* is the so-called effective coefficient of permeability of the porous material:

(16) $$B^{e}=\frac{\varepsilon }{\tau }\frac{d_{p}^{2}}{32}$$

It turns out that in multicomponent mixtures containing compounds with different molar masses, a total pressure gradient develops in the porous material [26,40]. This arises as a consequence of the difference in the Knudsen diffusion rates of the individual components. For this reason, the definition of molar flux density (10) needs to include an expression for viscous flow. This term introduces an additional mass transport mechanism which, unlike Knudsen diffusion, is not a separative mechanism with respect to individual substances.

An extremely significant step in the development of the Maxwell–Stefan formalism was its application to the modelling of the diffusive mass transport of species adsorbed on the internal surfaces of porous materials. A great contribution to the extension of the Maxwell–Stefan model to the description of surface diffusion was made by the works of Krishna [19,20,21]. It was proved that the framework of the Maxwell–Stefan model holds well also for process simulations and calculations of surface diffusion.

According to the mentioned suggestions of Krishna, the mass transport due to surface diffusion can be described by a system of equations:

(17) $$-\frac{\theta_{i}}{RT}\frac{d\mu_{i}^{s}}{dz}=\sum_{j=1,j\neq i}^{K}\frac{q_{j}^{*}\theta_{j}N_{i}^{s}-q_{i}^{*}\theta_{i}N_{j}^{s}}{\rho_{s}q_{i}^{*}q_{j}^{*}D_{ij}^{s}}+\frac{N_{i}^{s}}{\rho_{s}q_{i}^{*}D_{i}^{s}},\;(i=1,\;2,\ldots ,K)$$

After assuming thermodynamic equilibrium (4), the expression for the vector of molar flux densities, **N***^s^* is obtained:

(18) $$N^{s}=-\rho_{s}q^{*}\cdot {\left[B^{s}\right]}^{-1}\cdot \Gamma \cdot \frac{d\theta }{dz}$$

where matrix q^*^ is given by:

(19) $$q^{*}=\left[\begin{array}{cccc} q_{1}^{*} & 0 & .... & 0\\ 0 & q_{2}^{*} & .... & 0\\ .... & .... & .... & ....\\ 0 & 0 & .... & q_{K}^{*} \end{array}\right]$$

and

(20) $$B^{s}={\left\{B_{ij}^{s}\right\}}_{K\times K}={\left[\begin{array}{cc} \sum_{\begin{array}{l} k=1\\ k\neq i \end{array}}^{K}\frac{\theta_{k}}{D_{ik}^{s}}+\frac{1}{D_{i}^{s}}; & \left(i=j\right)\\ -\frac{\theta_{i}}{D_{ij}^{s}}; & \left(i\neq j\right) \end{array}\right]}_{K\times K}$$

The expression for **N***^s^* similarly to **N***^p^* appears in Equation (1). The values of the thermodynamic factors are calculated from the following relationship:

(21) $$\Gamma_{ij}=\frac{\theta_{i}}{p_{i}}\frac{\partial p_{i}}{\partial \theta_{j}}=\frac{q_{j}^{*}}{q_{i}^{*}}\frac{q_{i}}{p_{i}}\frac{\partial p_{i}}{\partial q_{j}}$$

It should be remarked that the information about the thermodynamic equilibrium (4) is necessary only when surface diffusion occurs. Moreover, the adoption of model (18) implies the need to determine inverse functions with respect to isotherms (5), i.e.,

(22) $$p_{i}=f_{i}^{-1}(q)\;\mathrm{or}\;p_{i}=g_{i}^{-1}(\theta ),\;T=\mathrm{const},\;(i=1,\;2,\ldots ,K)$$

This factor makes computations more difficult, especially when functions (5) are not given explicitly, e.g., when *q_i_* values are derived from numerical calculations e.g., according to ideal adsorption solution theory (IAST), real adsorption solution theory (RAST) [17] or are obtained on the basis of vacancy solution theory (VST) or from molecular simulations. For this reason, an alternative model, which does not contain a matrix of thermodynamic factors **Γ**, is presented below, and then a method for the determination of steady states for the discussed complex multicomponent diffusion is proposed.

### 2.2. An Alternative Model of Surface Diffusion

It can be easily shown that for the surface diffusion of a single species (*K* = 1), its molar flux density is:

(23) $$N_{1}^{s}=-\rho_{s}q_{1}^{*}D_{1}^{s}\frac{\phi (p_{1})}{p_{1}}\frac{dp_{1}}{dz}=-\rho_{s}D_{1}^{s}\frac{q_{1}(p_{1})}{p_{1}}\frac{dp_{1}}{dz}\;\left[\frac{\mathrm{mol}}{m^{2}s}\right]$$

After applying the Maxwell–Stefan formalism, one can derive the formula for surface diffusion for *K* components:

(24) $$-\rho_{s}\frac{q_{i}}{p_{i}}\frac{dp_{i}}{dz}=\sum_{j=1,j\neq i}^{K}\frac{q_{j}N_{i}^{s}-q_{i}N_{j}^{s}}{q_{j}^{*}D_{ij}^{s}}+\frac{N_{i}^{s}}{D_{i}^{s}},\;(i=1,\;2,\ldots ,K)$$

In matrix notation we have:

(25) $$N^{s}=-\rho_{s}{\left[B^{s}\right]}^{-1}\Delta \frac{dp}{dz}$$

After utilizing expression (9) in Equation (25) one gets:

(26) $$N^{s}=-\rho_{s}\cdot {\left[B^{s}\right]}^{-1}\cdot \Delta \cdot {\left[J\right]}^{-1}\frac{dq}{dz}$$

where **B***^s^* matrix determines surface diffusion:

(27) $$B^{s}={\left\{b_{ij}\right\}}_{K\times K}={\left[\begin{array}{cc} \sum_{\begin{array}{l} k=1\\ k\neq i \end{array}}^{K}\frac{q_{k}}{q_{k}^{*}D_{ik}^{s}}+\frac{1}{D_{i}^{s}}\;; & \left(i=j\right)\\ -\frac{q_{i}}{q_{j}^{*}D_{ij}^{s}}; & \left(i\neq j\right) \end{array}\right]}_{K\times K}$$

The matrix **Δ** present in Equations (25) and (26) has a diagonal form

(28) $$\Delta =\left[\begin{array}{cccc} \frac{q_{1}(p)}{p_{1}} & 0 & ..... & 0\\ 0 & \frac{q_{2}(p)}{p_{2}} & ..... & 0\\ ..... & ..... & ..... & .....\\ 0 & 0 & ..... & \frac{q_{K}(p)}{p_{K}} \end{array}\right]$$

The determination of the inverse of the matrix **Δ** is therefore an elementary problem. In surface diffusion models, there are coefficients of two types. They are the diffusion coefficients of the given *i-*th component $D_{i}^{s}$ and the counter-sorption diffusivities $D_{ij}^{s}$ between component A*_i_* and component A*_j_*. In this paper, the effect of the fractional surface occupancy on the values of the surface diffusion coefficients is addressed based on the following relationship:

(29) $$D_{i}^{s}=D_{i}^{s}(0)\cdot f(\theta_{t}),\;f(\theta_{t})={\left(1-\theta_{t}\right)}^{\alpha },\;\theta_{t}=\sum_{i}\theta_{i}$$

where $D_{i}^{s}(0)$ is the diffusion coefficient corresponding to zero coverage, i.e., θ*_t_* = 0 and α is the degree of confinement. Furthermore, the following formula holds:

(30) $$\underset{\alpha \to 0}{\lim}D_{i}^{s}=D_{i}^{s}(0)$$

The Maxwell–Stefan counter-sorption diffusivities were calculated according to the recommendations available in the literature [22]:

(31) $$\ln D_{ij}^{s}=\frac{\theta_{i}}{\theta_{i}+\theta_{j}}\ln D_{i}^{s}+\frac{\theta_{j}}{\theta_{i}+\theta_{j}}\ln D_{j}^{s}$$

### 2.3. Method for Determining Steady States

Mathematical formalism implies that the determination of the steady state should consist in taking the partial pressure derivatives with respect to time in Equation (8) to be equal to zero and solving the resulting system of equations. Following in this manner, from Equations (1), (10) and (25) the following formula will be obtained:

(32) $$\frac{1}{z^{m}}\frac{d}{dz}\left\{\frac{z^{m}}{RT}\left[{\left[B^{p}\right]}^{-1}\frac{dp}{dz}+\frac{B^{e}}{\eta }\frac{dp}{dz}{\left[B^{p}\right]}^{-1}E\cdot p\right]+z^{m}(1-\varepsilon )\rho_{s}{\left[B^{s}\right]}^{-1}\Delta \frac{dp}{dz}\right\}=0$$

It is a system of *K* second order ordinary differential equations due to *K* partial pressures *p_i_* (*i* = 1, 2, …, *K*). With given values of partial pressures for *z* = 0 and for *z* = *L* these equations can be solved obtaining *K* functions *p_i_*(*z*). Equation (32) was obtained assuming thermodynamic equilibrium between the gas and the adsorbed phase. Therefore knowing isotherms *q_i_*(**p**) one can calculate functions *q_i_* (*z*) for *i* = 1, 2, …, *K*.

Since the transport of mass in the pores and via surface diffusion are parallel mechanisms, both of these diffusion routes add up algebraically and give the total molar flux density per unit cross section. Due to the assumption of interphase thermodynamic equilibrium (5) and the resulting Equation (7), another approach for the determination of steady states can be proposed. To the best of the authors’ knowledge, such a method has not yet been published. The proposed method takes advantage of the transformed Equations (10) and (26). We illustrate it here using examples of diffusion through flat or cylindrical membranes of small thickness *L* in comparison with the membrane radius. In these cases, the cylindrical membrane can be treated as flat and one can assume *m* = 0. Then, the following system of 2 × *K* nonlinear equations is obtained that needs to be solved:

(33) $$\frac{dp}{d\xi }=-LRT\cdot B^{p}(p)\cdot N^{p}-\frac{B^{e}}{\eta }\frac{dp}{d\xi }E\cdot p$$

(34) $$\frac{dq}{d\xi }=-(1-\varepsilon )\frac{L}{\rho_{s}}J(p)\cdot \Delta^{-1}B^{s}[q(p)]\cdot N^{s},\;\xi =\frac{z}{L}\in [0,\;1]$$

where ξ is a dimensionless spatial coordinate. The pore volume fraction has been explained previously (Equations (14)–(16)). The expression **B***^s^* [**q**(**p**)] indicates that the values of **q** in the matrix **B***^s^* (Equation (27)) are determined using the partial pressures calculated from Equation (33). In Equation (34) the matrix of thermodynamic factors **Γ** does not appear, and the only matrix to be inverted is the diagonal matrix **Δ**. Information about the adsorption equilibrium is incorporated in the matrices **J** and **Δ**. Using the mathematical formalism, we state that a system of *K* second order differential Equation (32) correspond to 2 × *K* first order differential Equations (33) and (34).

There are boundary conditions associated with the systems of differential Equations (33) and (34). Their form depends on the mode in which the process is carried out. Let us assume that the boundary conditions are of the first type, i.e.,

(35) $$p(0)=p_{0}$$

(36) $$p(1)=p_{L}$$

(37) $$q(0)=q_{0}$$

(38) $$q(1)=q_{L}$$

The contribution of the external resistance to mass transport, i.e., from the gas to the membrane surface, decreases with increasing membrane thickness *L*. For a sufficiently large thickness *L* almost all the resistance to mass transport is localized in the membrane.

The method for determining the steady state of a specific diffusion system can be summarized by the following algorithm:

(a) adopt tentative values of **N***^p^* and **N***^s^*;
(b) assume the boundary conditions (35), (37) for ξ = 0;
(c) integrate systems of ordinary differential Equations (33) and (34) from ξ = 0 to ξ = 1;
(d) verify the fulfilment of the boundary conditions (36), (38) for ξ = 1;
(e) if the boundary conditions are not met, improve the values of **N***^p^* and **N***^s^* e.g., with the help of Newton’s overriding algorithm and return to point (b);
(f) if the boundary conditions are met, it means that the profiles of all 2 × *K* variables, i.e., **p**(ξ) and **q**(ξ) = **q** [**p**(ξ)] and the molar flux densities corresponding to pore diffusion **N***^p^* and surface diffusion **N***^s^* describe the determined steady state. Thence it is possible to calculate **N***_tot_* = **N***^p^* + **N***^s^*.

From the above algorithm, it follows that the determination of the steady state of the diffusion process under consideration reduces to solving a system of 2 × *K* algebraic equations, which can be written as:

(39) $$F(x,\lambda )=0,\;x=[N^{p},\;N^{s}]\in {ℜ}_{+}^{2K}$$

where **λ** is a vector of model parameters.

The steady-state characteristics of considered diffusion processes include concentration profiles in the gas **y**(ξ) and in the solid **θ**(ξ), as well as molar flux densities **N***^p^*, **N***^s^*, **N***_tot_*. The above algorithm illustrates how to determine all these quantities.

The systems of differential Equations (33) and (34) with boundary conditions (35)–(38) constitute a mathematical model of steady-state complex multicomponent diffusion. On the basis of this model, process analysis can be performed and the influence of the individual constituents, i.e., molecular and Knudsen diffusion, viscous flow and surface diffusion, can be assessed depending on the process conditions. Nevertheless, in order to assess the validity of the model (33)–(38) its reliability should be verified. The method for determining steady states was verified by comparing the results obtained from Equations (33) and (34) with the results obtained according to the dynamic model (8) for sufficiently long time *t*.

Let λ*_k_* be the *k*^th^ element of the vector of parameters **λ**. Applying the continuation of solutions of the system of Equations (39) with respect to this chosen parameter, one gets a branch of steady states. The use of a continuation algorithm, e.g., the local parameterization method [41], gives the possibility to determine the parametric dependence of the diffusion model (33)–(38).

## 3. Results and Discussion

The following results and discussions are aligned to the two objectives of this study. In particular, they are:

(a) To propose a mathematical model for the determination of steady states of complex multicomponent diffusion;
(b) To verify the method.

The solution of the steady state diffusion model (33)-(38) is the set of 2 × *K* functions:

(40) $$\left\{p_{i}(z),\;q_{i}(z)\}\;,\;(i=1,\;2,\ldots ,K)\;,\;z\in [0,\;1\right]$$

and the vectors **N***^p^* and **N***^s^*. Based on (40) it is possible to calculate:

(41) $$y_{i}(z)=\frac{p_{i}(z)}{p(z)},\;\theta_{i}(z)=\frac{q_{i}(z)}{q_{i}^{*}},\;(i=1,\;2,\ldots ,K)\;,\;z\in [0,\;1]$$

The validity of the steady-state model (33)–(38) can be verified by comparing the functions (40) and (41) obtained from this model with the profiles *p_i_*(*z*, *t*) and *q_i_*(*z*, *t*) derived from the solution of Equation (8) for *t* → ∞, and in practical calculations for sufficiently large values of time *t*.

### 3.1. Selection of Diffusion Systems and Process Parameters

In order to illustrate the discussed method for the determination of steady states, two diffusion systems were considered, i.e.,:

(a) diffusion of the ternary solution {A_1_, A_2_, A_3_} = {CH_4_, CO_2_, N_2_} through microporous activated carbon (set “I”);
(b) diffusion of the ternary solution {A_1_, A_2_, A_3_} = {CO_2_, C_3_H_8_, N_2_} through microporous glass Vycor (set “II”).

In the diffusion system “I”, all three components are adsorbed on the surface of the solid, while in the diffusion system “II”, the component A_3_ is assumed to be an inert not subject to adsorption. The structural parameters of the porous solids are given in Table 1. It was assumed that diffusion occurs through a flat membrane, as in a double-sided open Wicke-Kallenbach cell.

The physicochemical parameter values provided in Table 2 were used for process calculations.

### 3.2. Thermodynamic Equilibrium in Selected Diffusion Systems

Gas–solid interphase multicomponent equilibria were calculated according to the IAST (ideal adsorption solution theory) model [17,42,43]. Both IAS and RAS methods allow to determine multicomponent equilibria from isotherms or measurement results for pure components that are adsorbed on the same solid. This provides equilibrium concentrations for every given composition *y_i_*(*z*), (*i* = 1,2, …, *K*).

When the isotherms of pure components A*_i_* are given, then the calculation of *q_i_* = *f_i_*(*p*_1_, …, *p_K_*), (*i* = 1, …, *K*) can be obtained by the following algorithm:

(1) Assume the total pressure *p*, the gas phase composition *y_i_* and calculate the partial pressures *p_i_* = *py_i_*.
(2) Calculate the approximate value of the reduced spreading pressure η using the formula.

(42) $$\eta_{0}=\sum_{i=1}^{K}y_{i}\int_{0}^{p}\frac{q_{i}^{\circ }(p_{i})}{p_{i}}dp_{i}$$

In the specific case when the isotherms of pure substances are given by the Langmuir equations, one obtains the analytical expression

(43) $$\eta_{0}={\bar{q}}^{*}\ln\left(1+\sum_{i=1}^{K}b_{i}^{\circ }p_{i}\right),\;{\bar{q}}^{*}=\sum_{i}^{K}y_{i}q_{i}^{\circ *}$$

(3) Estimate the approximate values of the hypothetical pressures $p_{i}^{\circ }$ as
  (44) $$p_{i}^{\circ }=py_{i}\;\mathrm{or}\;p_{i}^{\circ }=\frac{1}{b_{i}}\sum_{j=1}^{K}b_{j}^{\circ }p_{j}\;\mathrm{for}\;\mathrm{Langsmuir}\;\mathrm{isotherms},\;(i=1,\;2,\ldots ,\;K)$$
(4) Solve a system of *K* + 1 algebraic equations with respect to η and $p_{i}^{\circ },\;(i=1,\ldots ,K)$
  (45) $$f(\eta )=\sum_{i=1}^{K}\frac{py_{i}}{p_{i}^{\circ }(\eta )}-1=0,\;\eta =\frac{a\pi }{RT}$$
  (46) $$\int_{0}^{p_{i}^{\circ }}\frac{q_{i}^{\circ }(p_{i})}{p_{i}}dp_{i}-\eta =0,\;(i=1,\;2,\ldots ,K)$$

For the particular case of pure substance isotherms given by the Langmuir formula, instead of Equation (46) we obtain:

(47) $$\eta -q_{i}^{\circ *}\ln[1+b_{i}^{\circ }p_{i}^{\circ }(\eta )]=0,\;(i=1,\;2,\ldots ,K)$$

(5) Calculate the solid phase concentrations *q_i_* using the relationship
  (48) $$x_{i}=\frac{py_{i}}{p_{i}^{\circ }(\eta )}$$
  (49) $$\frac{1}{q_{t}}=\sum_{i=1}^{K}\frac{x_{i}}{q_{i}^{\circ }},\;q_{i}^{\circ }=q_{i}^{\circ *}\frac{b_{i}^{\circ }p_{i}^{\circ }}{1+b_{i}^{\circ }p_{i}^{\circ }}$$
  (50) $$q_{i}=q_{t}x_{i},\;q_{t}=\sum_{i=1}^{K}q_{i}$$

The values *q_i_* = *f_i_* (*p*_1_, …, *p_K_*) calculated according to the algorithm (42)–(50) can be used directly to determine the steady states of a given complex diffusion problem according to the method discussed above. In this paper it was hypothesized that these results can be approximated by a chosen isotherm model, e.g., by the extended Langmuir model:

(51) $$q_{i}=q_{i}^{*}\frac{b_{i}p_{i}}{1+\sum b_{i}p_{i}}=q_{i}^{\circ *}\frac{b_{i}p_{i}}{1+\sum b_{i}p_{i}},\;(i=1,\;2,\ldots ,\;K)$$

The aforementioned hypothesis and the correctness of approximation (51) was verified computationally on the basis of previously assumed gaseous solutions. More precisely, the application of the extended Langmuir model is thermodynamically justified when the saturation concentrations of all A*_i_* components are the same. However, it turns out that it can also be used when the mentioned assumption is not fulfilled [25].

The work by van den Broeke and Krishna [22] gives the parameters of the Langmuir isotherms for the pure components A_1_ = CH_4_, A_2_ = CO_2_, A_3_ = N_2_. The values of these parameters for Kureha microporous activated carbon at temperature *T* = 345 K are given in Table 3. Equilibrium data for the diffusion system {A_1_, A_2_, A_3_} = {CO_2_, C_3_H_8_, N_2_} are provided in Table 4 [25].

It follows from the algorithm (42)–(50) that the values of parameters *b_i_* (*i* = 1, 2, 3) for mixtures are dependent on the composition of the gas phase *y_i_*. More specifically, when determining the steady states according to the model (33)–(34), one should use the algorithm (42)–(50) repeatedly for each set of values {*p*_1_, *p*_2_, …, *p_K_*}*_m_*. Let us observe that, according to the extended Langmuir model, one has:

(52) $$\frac{q_{i}}{q_{i}^{*}b_{i}p_{i}}-\frac{1}{1+\sum b_{i}p_{i}}=0,\;(i=1,\;2,\ldots ,\;K)$$

**Figure 2:**
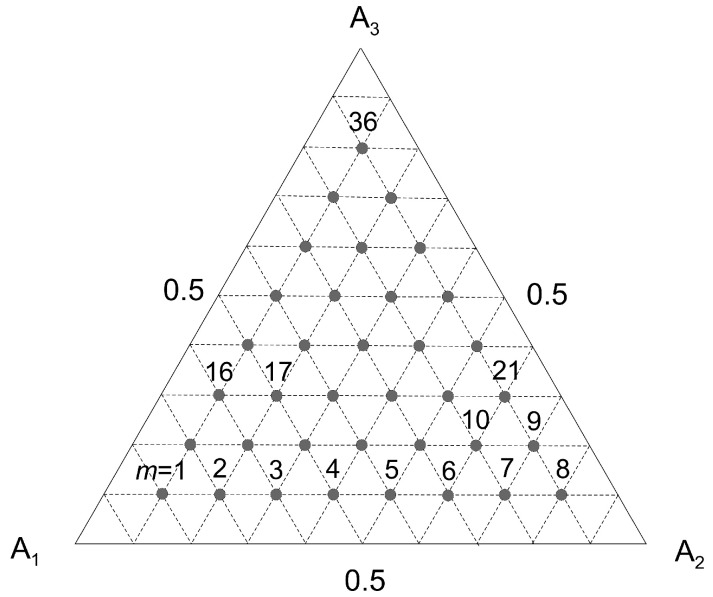
Graphical illustration of the set of values {*y*_1_, *y*_2_, *y*_3_}*_m_* adopted to determine the average values of parameters *b_i_* for ternary gaseous mixtures (*m* indicates the set number).

The second term of the left-hand side of Equation (52) has the same value for each component A*_i_*. Once the concentration *q_i_* is calculated according to the algorithm (42)–(50), one can solve a system *K* of algebraic Equation (52) with respect to *b_i_*. For *K* = 3 one obtains *M* sets of values of {*b*_1_, *b*_2_, *b*_3_}*_m_*, (*m* = 1, 2, …, *M*) where *M* is the number of data sets adopted in the calculations (Figure 2). In this work it is proposed to average these values for all possible gas phase compositions. To test this hypothesis, calculations of parameters *b_i_* were performed for the molar fractions of the gas phase indicated in Figure 2. The average values of *b_i_* were subsequently determined on this basis and adopted in the extended Langmuir model (51). The result was:

(a) for data set no. “I”: *b*_1_ = 2.719 × 10^−6^; *b*_2_ = 3.106 × 10^−6^; *b*_3_ = 1.952 × 10^−6^ Pa^−1^;
(b) for data set no. “II”: *b*_1_ = 2.206 × 10^−6^; *b*_2_ = 6.394 × 10^−6^ Pa^−1^.

**Figure 3:**
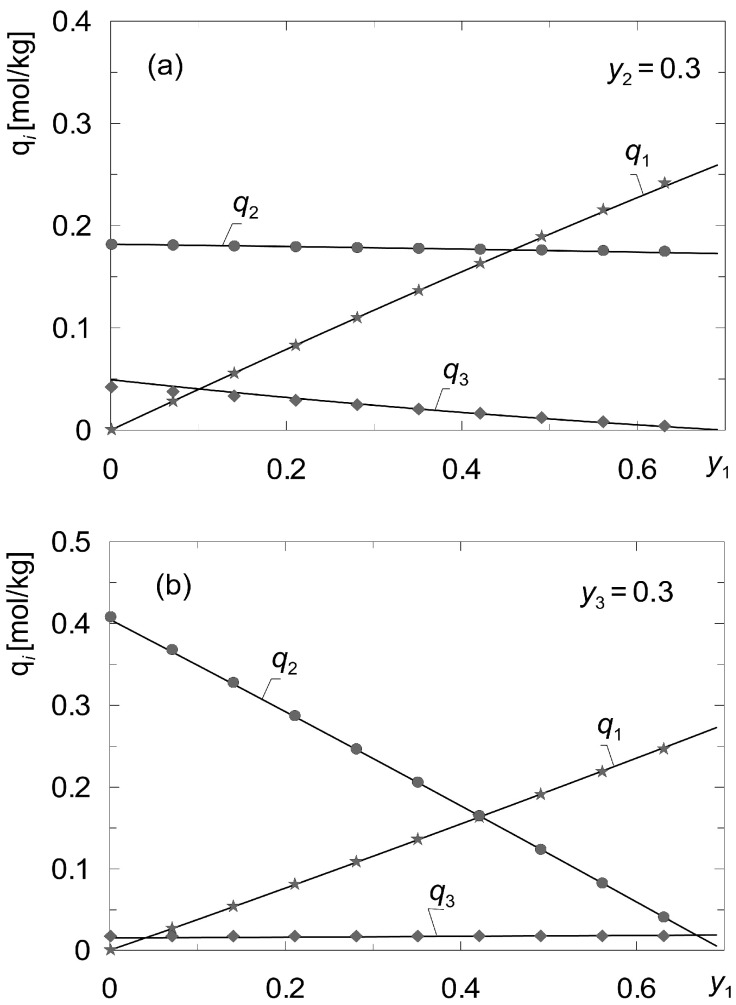
Comparison of the values of concentrations *q_i_* calculated according to the local gas composition {*y*_1_, *y*_2_, *y*_3_}*_m_* with those calculated from the average values of *b_i_* for the mixtures {A_1_, A_2_, A_3_} = {CH_4_, CO_2_, N_2_} according to the data set “I” (Table 3); (**a**) fixed *y*_2_ = 0.3; (**b**) fixed *y*_3_ = 0.3.

Figure 3 shows a comparison of the concentration values *q_i_* calculated based on the local gas composition {*y*_1_, *y*_2_, *y*_3_}*_m_* with those calculated based on the average *b_i_* for the gaseous solutions {CH_4_, CO_2_, N_2_}. As it can be observed, the proposed method proved to be effective. In the general case it can be applied when the values of {*b*_1_, *b*_2_, …, *b_K_*}*_m_*, (*m* = 1, 2, …, *M*) do not change significantly with the composition of the gas phase *y_i_* (*i* = 1, 2, …, *K*).

### 3.3. Verification of the Steady State Model

By solving the equations of the steady-state model (33), (34) one obtains functions *p_i_*(*z*), *q_i_*(*z*) and molar flux densities of individual components resulting from diffusion in pores and surface diffusion. This is an advantage of the presented method because it allows to determine the influence of model parameters and process conditions on the contributions of the above-mentioned molar fluxes, i.e., **N***^p^* and **N***^s^* in total fluxes **N***_tot_*.

**Figure 4:**
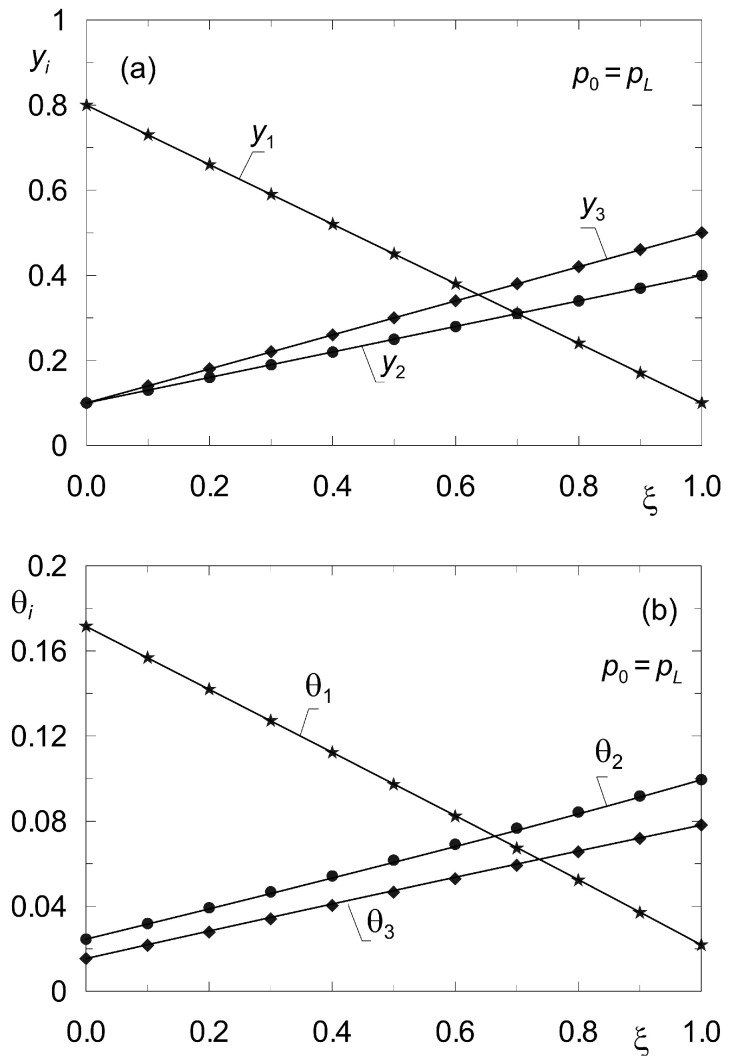
Profiles of molar fractions *y_i_*(*z*) (**a**) and fractional surface occupancies θ*_i_*(*z*) (**b**) obtained from the steady-state model (33), (34) (solid lines) and from the dynamic model (8) (point symbols) for complex ternary diffusion {CH_4_, CO_2_, N_2_} under isobaric conditions (*p*_0_ = *p_L_* = 10^5^ Pa) according to data set “I”. All components are subject to adsorption.

The summary collected in Table 5 gives algebraic values of molar flux densities obtained according to model (33), (34) for conditions corresponding to the profiles in Figure 4.

A well-known approach to verifying the validity of a steady-state model is to compare the results obtained using this model with the results obtained from the dynamic model for sufficiently long times. In the case of any newly proposed algorithm for the determination of steady states, such a procedure allows to prove its validity. It should bed noted that in this case, validation of the model (33), (34) consists in comparing the concentration distributions, i.e., the functions **y**(ξ) and **θ**(ξ) obtained from the system of *K* Equation (32) for a sufficiently long time *t* with the results obtained from the two systems (33), (34) with a total of 2*K* equations.

Figure 4 shows the distributions of molar fractions in the gas phase *y_i_* and fractional surface occupancy θ*_i_* obtained from the model (33), (34) for complex ternary diffusion under isobaric conditions according to the data of set “I” (Table 2). The solid lines originate from the solution of the boundary value problem (33) (38) with the method of shooting. Point symbols were obtained from dynamic simulations according to model (8) for sufficiently long time *t* > 500 s. The equations of diffusion dynamics (8) were solved by the method of lines [44].

**Figure 5:**
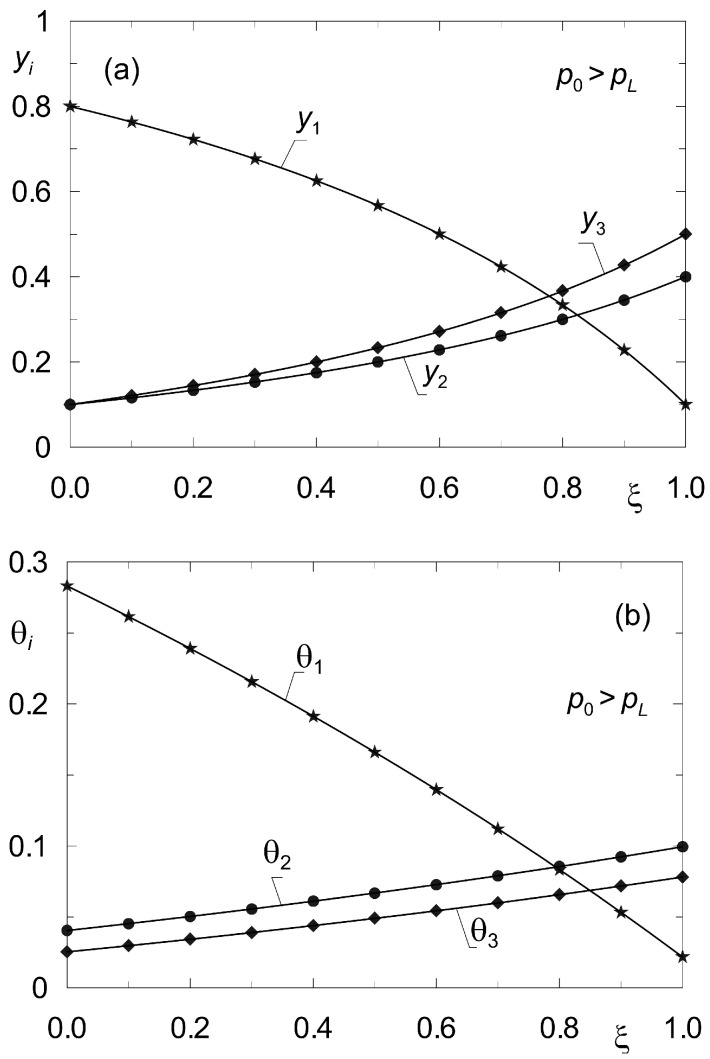
Profiles of molar fractions *y_i_*(*z*) (**a**) and fractional surface occupancies θ*_i_*(*z*) (**b**) obtained from the steady-state model (33)–(34) (solid lines) and from the dynamic model (8) (point symbols) for complex ternary diffusion under non-isobaric conditions (*p*_0_ = 2 × 10^5^ Pa; *p_L_* = 10^5^ Pa). All components are subject to adsorption.

For reference, a similar verification was performed in Figure 5 but for non-isobaric diffusion, when *p*_0_ > *p_L_*. In both cases, the verification of the steady-state model (33), (34) was found to be successful.

**Figure 6:**
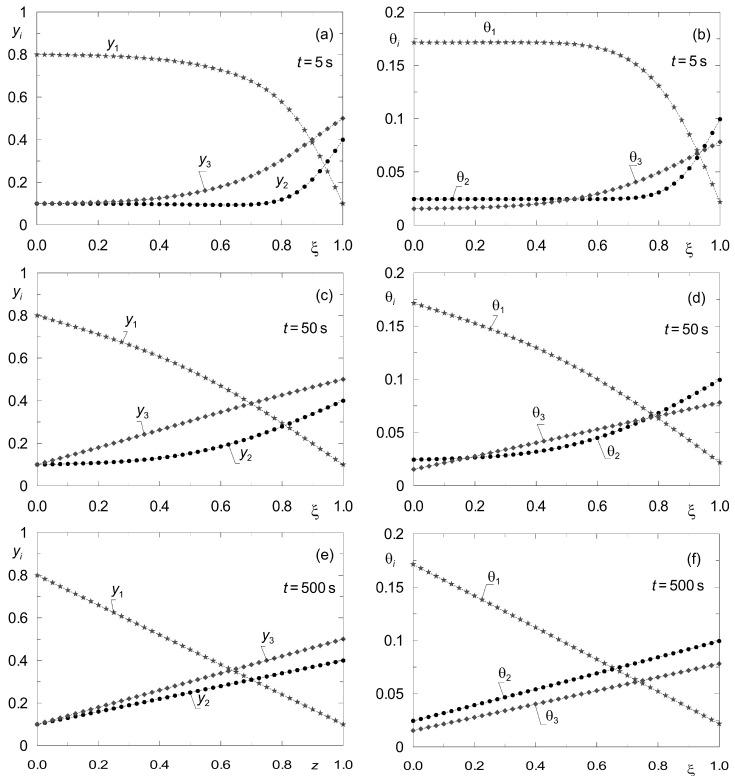
Illustration of the convergence of the transient profiles {*y_i_*(*z*, *t*), θ*_i_*(*z*, *t*)} obtained according to the dynamic model (8) to the steady state {*y_i_*(*z*), θ*_i_*(*z*)} for complex ternary diffusion under isobaric conditions for set “I”. All components are subject to adsorption. The point symbols correspond to the grid nodes in the line method (*p* = 10^5^ Pa); (**a**,**b**) for *t* = 5 s; (**c**,**d**) for *t* = 50 s; (**e**,**f**) for *t* = 500 s.

To determine the rate of convergence of the dynamic profiles *p_i_*(*z*, *t*), *q_i_*(*z*, *t*) or *y_i_*(*z*, *t*), θ*_i_*(*z*, *t*) to a given steady state, process simulations were performed for several values of time *t*. The results are shown in Figure 6. On this basis, the time *t* after which the profiles of the state variables *y_i_*(*z*) and θ*_i_*(*z*) obtained according to the two methods mentioned above can be compared was estimated.

The issues of complex diffusion discussed above concern processes in which all *K* components of the gas phase are adsorbed. In practice, however, we encounter processes in which there are inerts, which are not adsorbed, e.g., in gas cleaning processes. For inert components, surface diffusion does not occur. It turns out that the extension of the model (33), (34) to such processes does not pose any calculation difficulties.

**Figure 7:**
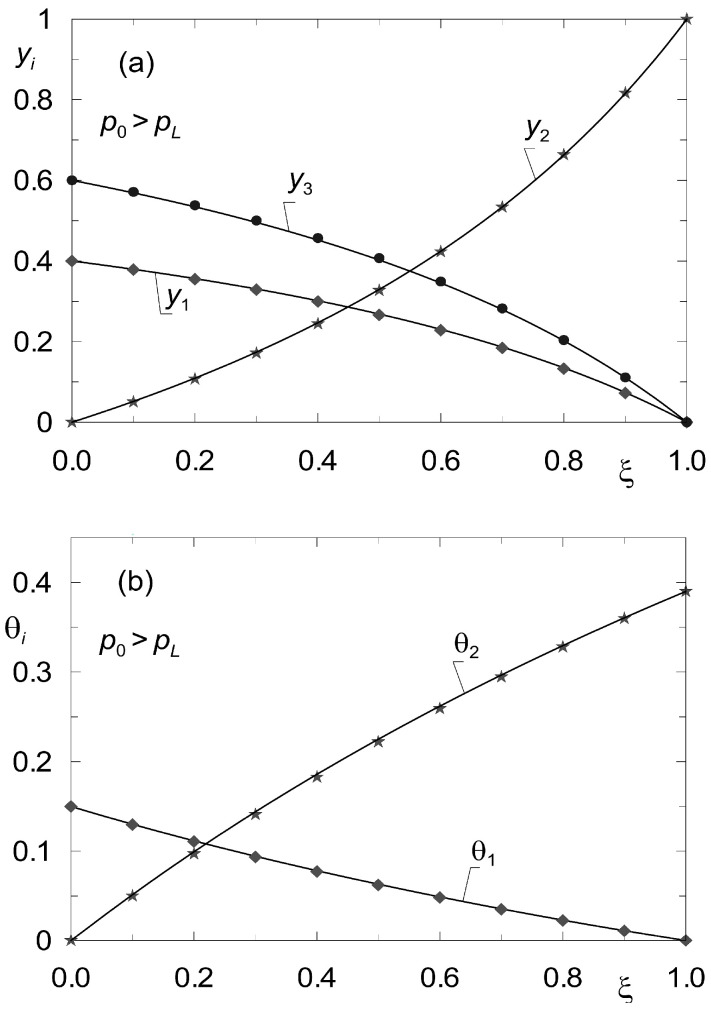
Profiles of molar fractions *y_i_*(*z*) (**a**) and fractional surface occupancies θ*_i_*(*z*) (**b**) obtained from the steady-state model (33)–(34) (solid lines) and from the dynamic model (8) (point symbols) for complex ternary diffusion under non-isobaric conditions (*p*_0_ = 2 × 10^5^ Pa; *p_L_* = 10^5^ Pa). Two components undergo adsorption, the third component is inert (set “II”).

**Figure 8:**
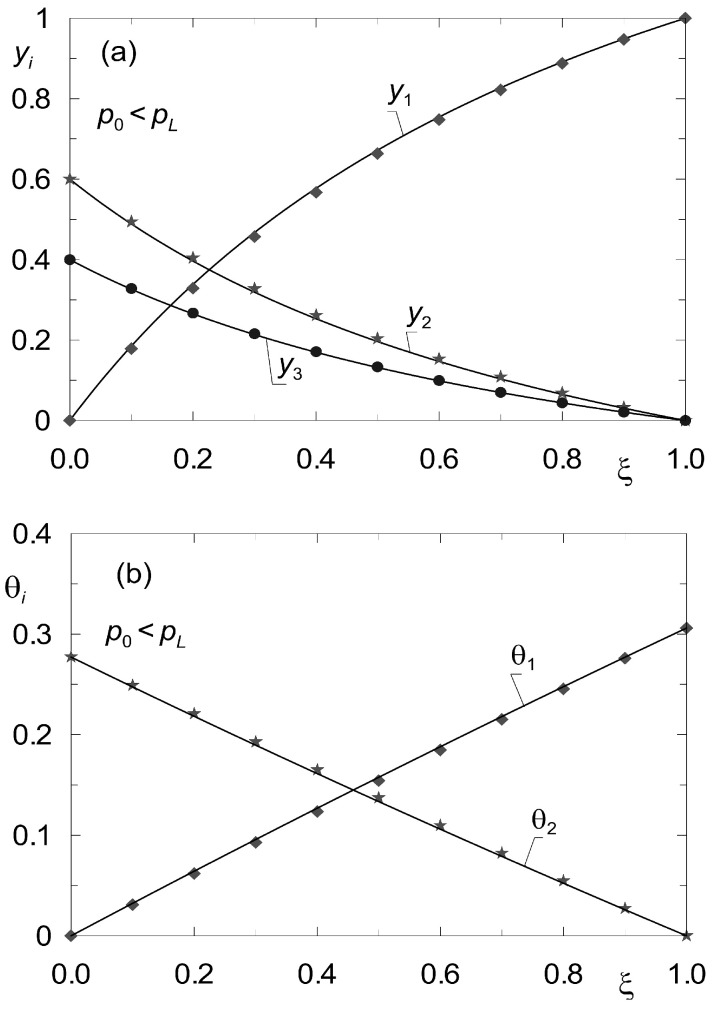
Profiles of molar fractions *y_i_*(*z*) (**a**) and fractional surface occupancies θ*_i_*(*z*) (**b**) obtained from the steady-state model (33)–(34) (solid lines) and from the dynamic model (8) (point symbols) for complex ternary diffusion under non-isobaric conditions (*p*_0_ = 10^5^ Pa; *p_L_* = 2 × 10^5^ Pa). Two components undergo adsorption, the third component is inert (set “II”).

Let *K_n_* be the number of components not being adsorbed. Then the determination of the steady state reduces to solving 2 × *K* – *K_n_* differential equations of the form analogous to (33), (34). The difference consists in the fact that instead of *K* Equation (34), *K* − *K_n_* equations describing surface diffusion must now be solved. For the sake of illustrating the discussed method in application to diffusion processes in the presence of inerts, data from the work of Tuchlenski and co-authors [25] were adopted. They are listed in Table 2 and Table 4 and labelled as set “II”. Process calculations were carried out for ternary non-isobaric diffusion in which the third component (A_3_) is treated as inert, which does not undergo adsorption. The simulation results are presented in Figure 7 and Figure 8. The state variable profiles in Figure 7 correspond to the process where *p*_0_ > *p_L_*, while Figure 8 illustrates the steady state reflecting the conditions where *p*_0_ < *p_L_*. The point symbols represent the results of verification of the steady state model (33), (34) by dynamic simulations.

## 4. Conclusions

The paper presents a general model describing the steady states of complex multicomponent diffusion in meso- and microporous materials. The model accounts for molecular diffusion, Knudsen diffusion, viscous flow and surface diffusion. A method of implementing this model to determine steady states is provided. The method is presented in an algorithmic form, which facilitates the development of numerical codes. The model (33), (34) was verified by comparing the state–state profiles with the state variable distributions obtained from dynamic model for sufficiently long process times.

The presented steady-state model does not require the calculation of a matrix of thermodynamic factors, which is an important advantage when using analytically irreversible sorption isotherms, or those determined numerically.

Upon solving the equations of the steady-state model (33), (34), the distributions of the state variables, i.e., **p**(*z*, *t*), **q**(*z*, *t*) or **y**(*z*, *t*), **θ**(*z*, *t*), as well as the values of molar flux densities in the pores **N***^p^* and in the adsorbed phase **N***^s^* are obtained. This makes it possible to determine the influence of process conditions on the contributions of mass transfer in the pores and by surface diffusion, and thus to identify the process. Custom software codes developed in the Fortran language were used for the numerical simulations.

The proposed algorithm for the determination of steady states can be easily used for systems with any number of components and also allows taking into account the presence of inert gases, which are not subject to adsorption. For such cases, equations describing diffusion in pores (33) remain unchanged. Only the number of equations in system (34) describing surface diffusion decreases and is equal to the number of adsorbable components.

The study also provides a somewhat work-around but effective way of approximating isotherms for multicomponent equilibria using data for pure substances. The method consists of the appropriate selection of equilibrium values calculated according to the IAS method at selected nodes of concentration grids *y_i_*. The dimension of these grids depends on the number of active components and is equal to (*K* − *K_n_* − 1).

The presented model and method for the determination of steady states was applied to two examples. In the first one, the diffusion of three components, i.e., CH_4_, CO_2_ and N_2_ through microporous activated carbon was considered. In this process, all three components are adsorbed and have different saturation concentrations. Model (33)–(38) as applied to this process consists of three equations for the gas phase (33) and three equations for the adsorbed phase (34). In the second example, also the diffusion of three components, i.e., CO_2_, C_3_H_8_ and N_2_ through a microporous Vycor glass was analyzed. In this process only the first two components are adsorbed. Nitrogen is the non-adsorbable inert. Simulations were carried out for both isobaric and non-isobaric processes.

The proposed method for the determination of steady states in complex multicomponent mass transport in porous media is of a universal nature. It can be a useful tool for numerical simulations, for design calculations and for theoretical studies of such processes.

## Figures and Tables

**Table 1:** Structural parameters of solids.

Porous Material	ρ*_s_* (kg·m^−3^)	*d_p_* (m)	ε
Microporous activated carbon [22] (Set “I”)	2100	5.2 × 10^−10^	0.64
Vycor microporous glass [25] (Set “II”)	2057	4.7 × 10^−9^	0.284

**Table 2:** Values of parameters adopted for numerical simulations for two data sets.

Parameter	Set “I” [22]	Set “II” [25]	Value
*T*	345	343	K
*y*_01_	0.80	0.40	−
*y*_02_	0.10	0.00	−
*y_L_*_1_	0.10	0.00	−
*y_L_*_2_	0.40	0.10	−
$D_{12}$	1.1647× 10^−5^	1.193 × 10^−5^	m^2^⋅s^−1^
$D_{13}$	1.4095 × 10^−5^	2.109 × 10^−5^	m^2^⋅s^−1^
$D_{23}$	1.0650 ×10^−5^	1.495 × 10^−5^	m^2^⋅s^−1^
$D_{1}^{s}$	1.375 × 10^−10^	2.80 × 10^−9^	m^2^⋅s^−1^
$D_{2}^{s}$	5.819 × 10^−11^	4.00 × 10^−9^	m^2^⋅s^−1^
$D_{3}^{s}$	3.174 × 10^−10^	−	m^2^⋅s^−1^
$\eta_{1}^{\circ }$	1.248 × 10^−5^	1.696 × 10^−5^	Pa⋅s
$\eta_{2}^{\circ }$	1.809 × 10^−5^	9.464 × 10^−6^	Pa⋅s
$\eta_{3}^{\circ }$	1.968 × 10^−5^	1.936 × 10^−5^	Pa⋅s
*L*	10^−3^	10^−3^	m

**Table 3:** Langmuir model parameters of pure substances at temperature *T* = 345 K on activated carbon [22].

Quantity	A_1_ = CH_4_	A_2_ = CO_2_	A_3_ = N_2_
$q_{i}^{\circ *},\;\mathrm{mol}\cdot kg^{-3}$	1.8	2.4	0.38
$b_{i}^{\circ },\;{\mathrm{Pa}}^{-1}$	2.7 × 10^−6^	3.0 × 10^−6^	3.0 × 10^−6^

**Table 4:** Langmuir model parameters of pure substances at temperature *T* = 343 K on microporous Vycor glass [25].

Quantity	A_1_ = CO_2_	A_2_ = C_3_H_8_	A_3_ = N_2_
$q_{i}^{\circ *},\;\mathrm{mol}\cdot kg^{-3}$	1.531	0.3395	0
$b_{i}^{\circ },\;{\mathrm{Pa}}^{-1}$	1.91 × 10^−6^	7.14 × 10^−6^	0

**Table 5:** Algebraic values of molar flux densities obtained according to model (33), (34) for conditions corresponding to the profiles in Figure 4.

	A_1_ = CH_4_	A_2_ = CO_2_	A_3_ = N_2_
$N_{i}^{p}$ (mol⋅m^−2^⋅s^−1^)	7.103 × 10^−4^	−1.838 × 10^−4^	−3.072 × 10^−4^
$N_{i}^{s}$ (mol⋅m^−2^⋅s^−1^)	1.788 × 10^−4^	−5.252 × 10^−5^	−3.114 × 10^−5^
*N_tot_*_,*i*_ (mol⋅m^−2^⋅s^−1^)	8.891 × 10^−4^	−2.363 × 10^−4^	−3.383 × 10^−4^

## Data Availability

Data available on request.

## Nomenclature

*a*	specific area of porous solid (m^2^⋅kg^−1^)
A_i_	*i*-th component
*b_i_*	constant in Langmuir multicomponent isotherm (Pa^−1^)
$b_{i}^{\circ }$	constant in Langmuir isotherm for pure species (Pa^−1^)
B ^e^	effective permeability parameter (m^2^)
*d_p_*	pore diameter, (m)
*D_ij_*	binary Maxwell–Stefan diffusion coefficient in gas phase (m^2^⋅s^−1^)
$D_{i}^{s}$	Maxwell–Stefan surface diffusivity of unary *i*-th species (m^2^⋅s^−1^)
$D_{ij}^{s}$	Maxwell–Stefan counter-sorption diffusivity (m^2^⋅s^−1^)
*D_Kn,i_*	Knudsen diffusion coefficient of *i*-th species (m^2^⋅s^−1^)
*f_i_*	fugacity coefficient
He	Henry constant for adsorption (mol⋅kg^−1^⋅Pa^−1^)
*K*	number of species
*L*	thickness of porous membrane (m)
*m*	shape coefficient
*M_i_*	molar mass of species i (kg⋅mol^−1^)
**N** *^p^*	vector of pore molar fluxes (mol⋅m^−2^⋅s^−1^)
**N** *^s^*	vector of surface molar fluxes (mol⋅m^−2^⋅s^−1^)
**N** *_tot_*	vector of total diffusive molar fluxes (mol⋅m^−2^⋅s^−1^)
*p*	total pressure (Pa)
*p_i_*	partial pressure of *i*-th component (Pa)
*q_i_*	adsorbed phase concentration of *i*-th component (mol⋅kg^−1^)
**q** ^*^	vector of saturation capacities (mol⋅kg^−1^)
*R*	universal gas constant (J⋅mol^−1^⋅K^−1^)
*t*	time (s)
*T*	temperature (K)
*x_i_*	molar fraction of *i*-th component in adsorbed phase
*y_i_*	molar fraction of *i*-th component in gas phase
*z*	spatial coordinate (m)
Greek letters	
α	degree of confinement
γ_ι_	activity coefficient of *i*-th species
**Γ**	matrix of thermodynamic correction factors
ε	porosity
η	viscosity (Pa⋅s)
η	reduced spreading pressure (mol⋅kg^−1^)
θ*_ι_*	fractional surface occupancy of component *i*
**λ**	vector of model parameters
µ	chemical potential (J⋅mol^−1^)
ξ	dimensionless spatial coordinate
π	spreading pressure (J⋅m^−2^)
ρ*_σ_*	skeletal membrane density (kg⋅m^−3^)
τ	tortuosity
Superscripts	
*	equilibrium value
°	standard state or pure species
*e*	effective value
*g*	gas phase
*p*	pore diffusion
*s*	surface diffusion
Subscripts	
*i*	*i*-th species in mixture
*m*	*m*-th point onto selected lattice
*n*	nonadsorbable species
*s*	solid phase
*tot*	total molar flux
0, *L*	refer to *z* = 0 or *z* = *L*, respectively
